# An Atypical Cause of an Epiglottic Abscess

**DOI:** 10.1155/2019/9674852

**Published:** 2019-12-12

**Authors:** Muneera Rabeea, Hasan Al Ansari, Amal Al Abdulla

**Affiliations:** Bahrain Defence Force Royal Medical Services, Riffa, Bahrain

## Abstract

An epiglottic abscess is a rare complication of acute epiglottitis and is life threatening. We describe a case report of a diabetic adult male, who presented with an epiglottic abscess. Culture results showed *Aeromonas hydrophila*, *a*n organism rarely reported as a cause of acute epiglottitis. Early recognition can be lifesaving.

## 1. Background

An epiglottic abscess is a rare complication of acute epiglottitis that is characterized by supraglottic collection of pus [1]. Although acute epiglottitis is commonly seen in children [2], an epiglottic abscess tends to be a disease of adults [3]. A common causative organism is *Haemophilus influenzae* type B (HIB) [2], but due to HIB vaccines, the incidence of HIB epiglottitis has declined. Although HIB is a common causative organism in children, it is not the only causative organism in adults, and other pathogenic bacteria have been found, including *Klebsiella pneumoniae, Streptococcus pneumoniae*, *and Staphylococcus aureus* which may explain the rising incidence of acute epiglottitis in adults [3]. An epiglottic abscess requires urgent diagnosis and treatment as it is been documented to have a high mortality rate. We present a case of a diabetic patient with an atypical cause of an epiglottic abscess.

## 2. Case Presentation

A forty-eight-year-old Bahraini male, known case of diabetes mellitus, presented to the emergency department with a history of throat pain for one day, associated with dysphagia and dysphonia. The patient denied any history of seafood ingestion or immersive contact with freshwater.

Upon his arrival to the emergency department, he was not in respiratory distress. Vitals were as follows: BP 142/91 mmHg, HR 118 bpm, temp 37.1°C, RR 19 breaths/min, and SaO_2_ 98% on room air. Rest of physical examination was unremarkable.

Few hours later, he developed respiratory distress. ABC protocol commenced. Patient was intubated immediately in the emergency department. A bedside flexible laryngoscope demonstrated a diffusely oedematous obstructing epiglottis.

Laboratory investigations showed leukocytosis of 24.12 × 10^9^/L with 87% neutrophils and elevated C-reactive protein of 41.35 mg/L. Serology profile was negative for *Human Immunodeficiency Virus* (HIV).

**Figure 1:**
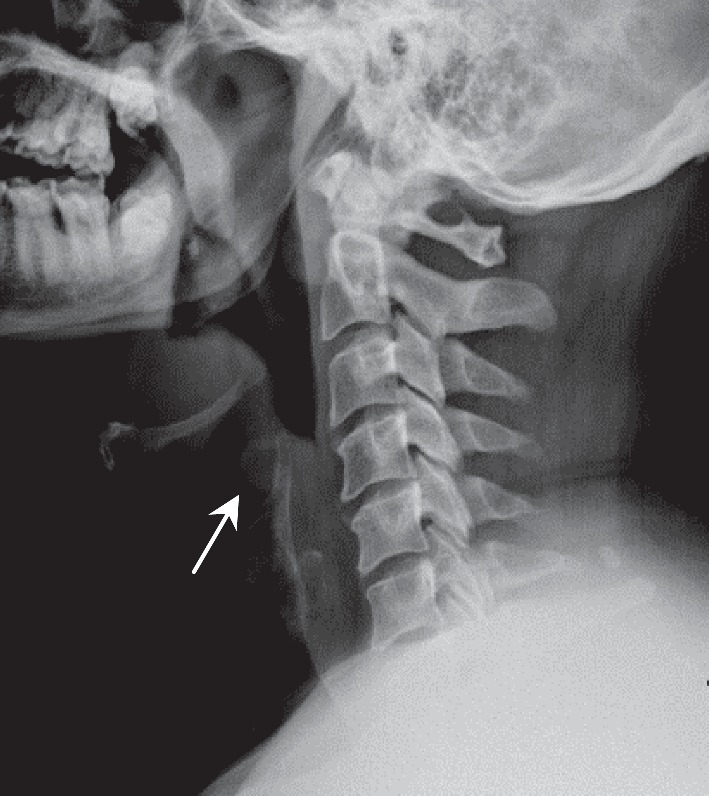
Radiography of lateral soft tissue neck.

Radiological investigations in the form of lateral soft tissue neck X-ray displayed the classical thumbprint sign appearance of epiglottitis (Figure 1).

### 2.1. Hospital Course

The patient was admitted in the intensive care unit under otolaryngology care with a provisional diagnosis of acute epiglottitis. He was managed conservatively with close airway monitoring, broad spectrum intravenous antibiotics, intravenous corticosteroids, racemic epinephrine, and antihistamine.

**Figure 2:**
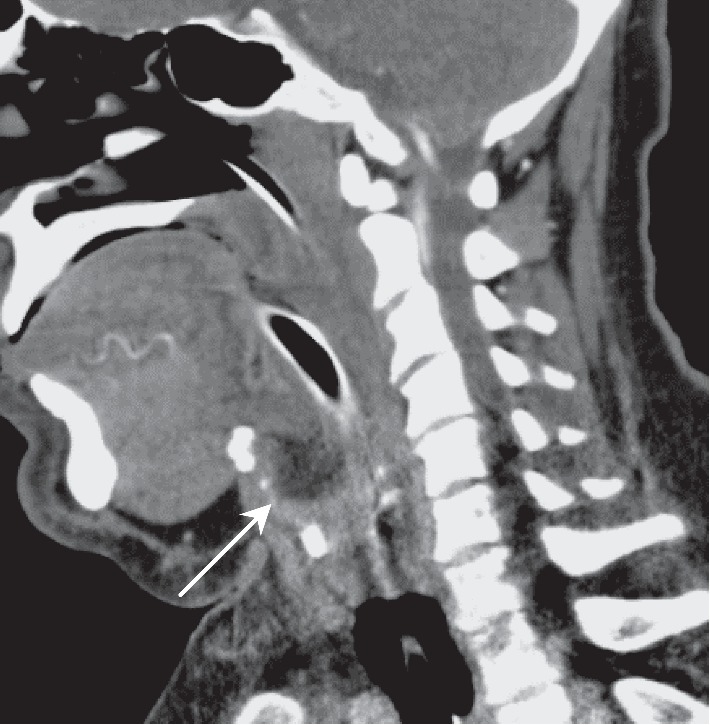
Sagittal view of CT neck with contrast demonstrating an extensive parapharyngeal soft tissue swelling and oedema, with an arrow pointing to the epiglottic abscess formation.

On day 5 of admission, flexible fiberoptic laryngoscopy revealed purulent drainage from the supraglottic region. Accordingly, a computed tomography (CT) neck with contrast was requested; findings were suggestive of an epiglottic abscess (Figure 2).

**Figure 3:**
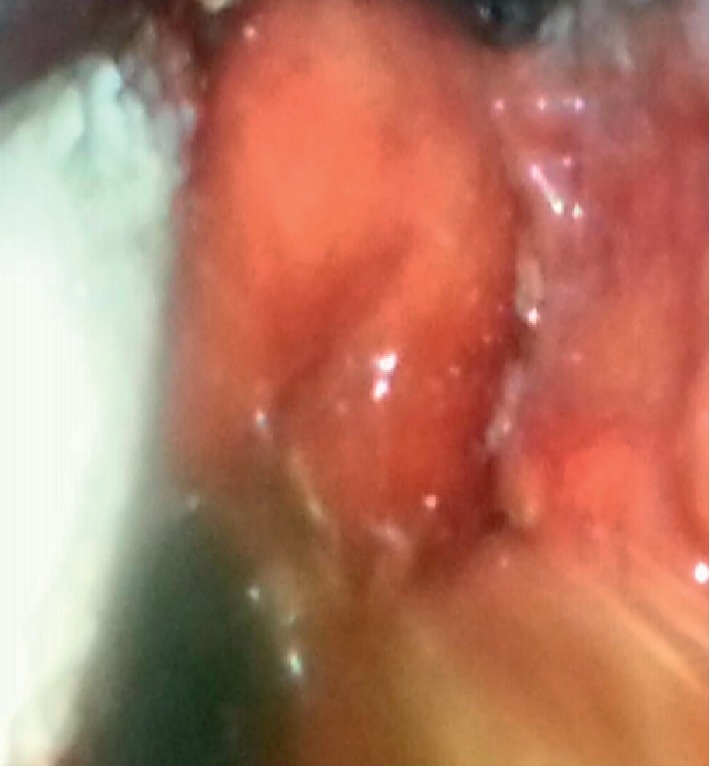
Intraoperative view showing an oedematous, congested epiglottis, draining pus from the inferior border of the left lateral glossoepiglottic fold.

The patient underwent upper rigid endoscopy under general anaesthesia to localise the site of pus drainage and aspirate the abscess. Pus was found draining from the inferior border of the left lateral glossoepiglottic fold (Figure 3). A swab was collected and sent for culture and sensitivity. No incision was necessary as the pus had already drained, and residual pus was aspirated using suction.

Postoperatively, the patient was extubated after a positive cuff leak test. Symptoms improved markedly, and he was discharged home the next day on oral antibiotic.

Patient was followed-up in the clinic one week after discharge. The patient was in good condition, had no complaints, and breathing and voice were normal. Flexible laryngoscopy showed a normal epiglottis, no pus, and normal patient airway mobile vocal cords.

Microbiology results showed a moderate growth of *Candida* and *Aeromonas hydrophila*.

## 3. Discussion

Epiglottic abscess is a rare complication of acute epiglottitis, and in the past, the most common causative organism was *Haemophilus influenzae* type B [4]. However, due to the HIB vaccine, other organisms have risen such as *Streptococcus pneumoniae, β-hemolytic streptococci, streptococcus pyogenes*, *and Staphylococcus aureus* [3, 5, 6]. In our patient, the causative organism was *Aerornonas hydrophila*. Apisarnthanarak et al. reported a case of necrotizing soft tissue infection and fasciitis resulting from acute epiglottitis caused by *Aeromonas hydrophila.* This case demonstrates the severity and virulence of the organism [7].

*Aeromonas hydrophila* is a Gram-negative facultative anaerobe, which is commonly found in either sewage or freshwater sources; in humans, its spread is usually via the faecal-oral route and predominantly causes diarrhoea and soft tissue infections [6]. Acute epiglottitis caused by *Aeromonas hydrophila* has rarely been reported in the literature [7] and has never been reported in the Kingdom of Bahrain [8]. *Aeromonas hydrophila* infection typically affects immunocompromised individuals who have been exposed to contaminated water or soil either through ingestion or a skin wound [7]. Wells et al. described a patient with chronic renal disease who developed parapharyngeal infection caused by *Aeromonas hydrophila* [9]. Both Berger et al. [3] and Ridgeway et al. [10] described diabetes mellitus as a risk factor for developing epiglottic abscess. Similarly, the patient described in our case report was a known case of diabetes mellitus and developed an epiglottic abscess caused by *Aeromonas hydrophila.*

An epiglottic abscess is usually a result of an infection of the epiglottis, and patients would initially complain of dysphagia and odynophagia followed by dysphonia and breathing difficulty. All patients are in danger of having rapid progression of airway compromise, and adults have an equal risk of rapid disease progression as in children. It is therefore critical to monitor the patient for oxygen desaturation and look for worsening stridor or if patient adapts a tripod position and drooling. Tracheostomy should be done to secure the airway if indicated [11].

Flexible laryngoscopy is an essential tool in assessing patients suspected of having epiglottitis or epiglottic abscess. This would allow a more accurate assessment of the airway and allow clinicians to determine the likelihood of impending airway obstruction. Radiological investigations are only to be done once the airway is secured or if the patient is deemed stable. A lateral plain radiograph may be obtained and may show the thumbprint sign; however, lateral neck radiograph has little diagnostic value in the absence of competent personnel. A CT scan of the neck is extremely valuable in assessing not only the airway but also in differentiating acute epiglottitis from epiglottic abscess [12].

The primary goal in managing a patient with epiglottic abscess is securing the airway. The airway is usually managed with endotracheal intubation or surgical tracheostomy under local anaesthesia, and intubation is preferred as tracheostomy has been linked to prolonged hospitalization and higher mortality [13]. Intubation should be done cautiously with minimal airway stimulation. The presence of an otolaryngologist is necessary for possible tracheostomy, in case of difficult intubation.

Once the airway is secured, the patient can be started on intravenous antibiotic preferably second or third generation cephalosporin combined with empiric therapy penicillinase-resistant penicillin. Corticosteroids may be used either intravenous or in aerosol form to reduce the resultant laryngeal oedema, airway compromise, and length of hospital stay. Other additional treatments include supplemental oxygen, intravenous fluids, and racemic epinephrine [14].

The definitive treatment of epiglottic abscess is drainage. A pus swab should be collected for culture and sensitivity to identify the causative organism and to target the antibiotic choice.

## 4. Conclusion

Early recognition and prompt management are lifesaving for patients presenting with epiglottic abscess. Although *Aeromonas hydrophila* is a rare causative organism, it should be considered one of the differential diagnosis of acute epiglottitis and epiglottic abscess.
